# Bladder Outlet Obstruction Due to Recurrent Complete Labial Fusion in Postmenopausal Woman—Case Report

**DOI:** 10.3390/diagnostics14232659

**Published:** 2024-11-25

**Authors:** Mihaela Camelia Tîrnovanu, Elena Cojocaru, Vlad Gabriel Tîrnovanu, Bogdan Florin Toma, Cristian Păvăloiu, Loredana Toma, Diana Elena Parteni, Ștefan Dragoș Tîrnovanu

**Affiliations:** 1 Department of Mother and Child Medicine, “Grigore T. Popa” University of Medicine and Pharmacy, 700115 Iasi, Romania; mihaela.tirnovanu@umfiasi.ro; 2 “Cuza Voda” Obstetrics-Gynecology Clinic Hospital, 700038 Iasi, Romania; bogdan-florin-sl-toma@d.umfiasi.ro (B.F.T.); cristian.pavaloiu@yahoo.com (C.P.); 3 Department of Morphofunctional Sciences I, “Grigore T. Popa” University of Medicine and Pharmacy, 700115 Iasi, Romania; parteni.diana-elena@d.umfiasi.ro; 4 “Saint Mary” Emergency Children’s Hospital, 700309 Iasi, Romania; 5 “St. Josef Hospital”, 65189 Wiesbaden, Germany; vlad.tirno@gmail.com; 6 Balneophysiokinetotherapy and Rehabilitation, “Grigore T. Popa” University of Medicine and Pharmacy, 700115 Iasi, Romania; loredana-toma@umfiasi.ro; 7 “Elena Doamna” Obstetrics-Gynecology Clinic Hospital, 700038 Iasi, Romania; 8 Department of Surgery II-Orthopedics and Traumatology, “Grigore T. Popa” University of Medicine and Pharmacy, 700115 Iasi, Romania; stefan-dragos.tirnovanu@umfiasi.ro; 9 “Saint Spiridon” County Emergency Clinical Hospital, 700111 Iasi, Romania

**Keywords:** labial fusion, bladder outlet obstruction, urinary retention, postmenopausal women, labial separation

## Abstract

Background: Labial fusion is a rare condition typically observed in prepubescent girls, but it can also occur in postmenopausal women due to estrogen deficiency. Methods: Our case report presents a unique instance of recurrent complete labial fusion causing bladder outlet obstruction in a postmenopausal woman. Results: The 84-year-old patient presented with dysuria and intermittent urine leakage. A physical examination revealed complete labial fusion with a small central opening. The diagnosis was confirmed through clinical evaluation, and surgical intervention was necessary to relieve the obstruction. Recurrent episodes followed despite a hormonal treatment with topical estrogen. Conclusions: This case highlights the importance of early diagnosis and the challenge of managing labial fusion in postmenopausal women, where recurrent episodes may complicate treatment. Surgical management, along with hormone therapy, remain essential for optimal outcomes. Further studies are needed to understand the recurrence mechanisms and to establish standardized treatment protocols.

## 1. Introduction

Labial adhesion, also known as labial synechiae or labial coalescence, refers to the complete or partial fusion of the labia minora at the midline through filiform or dense adhesions, forming a raphe [1]. It can be either congenital or acquired. In postmenopausal women, these adhesions may also affect the labia majora [1]. In adults, labial adhesion is a rare clinical condition, and the incidence is unknown, with only a limited number of cases documented in the literature. The incidence of labial adhesions in children is approximately 0.6–1.4%, while the incidence in the elderly population remains unclear [2]. According to Singh, the mean age among a group of six patients with labial adhesions was 76 years, with the ages ranging from 61 to 85 years [3].

The precise cause of labial adhesion is not fully understood. Because the underlying cause remains unclear, healthcare providers are uncertain regarding how to prevent labial adhesion. However, maintaining cleanliness in the area and monitoring for signs of irritation, such as redness, rashes, or pain, are advisable. In acquired cases, the potential underlying factors include estrogen deficiency, injuries, Behçet’s disease, Stevens–Johnson syndrome, mucous membrane pemphigoid, and graft-versus-host disease [4]. Additional precipitating factors may include chronic inflammation, seborrheic dermatitis, eczema, recurrent urinary tract infections, lichen planus, lichen sclerosus, urinary incontinence, and the absence of sexual activity, particularly in postmenopausal women [4]. Furthermore, postmenopausal women with orthopedic hip or neurologic lower extremity issues are generally at higher risk due to reduced or absent sexual activity and poor perineal hygiene [5].

## 2. Case Report

**Figure 1:**
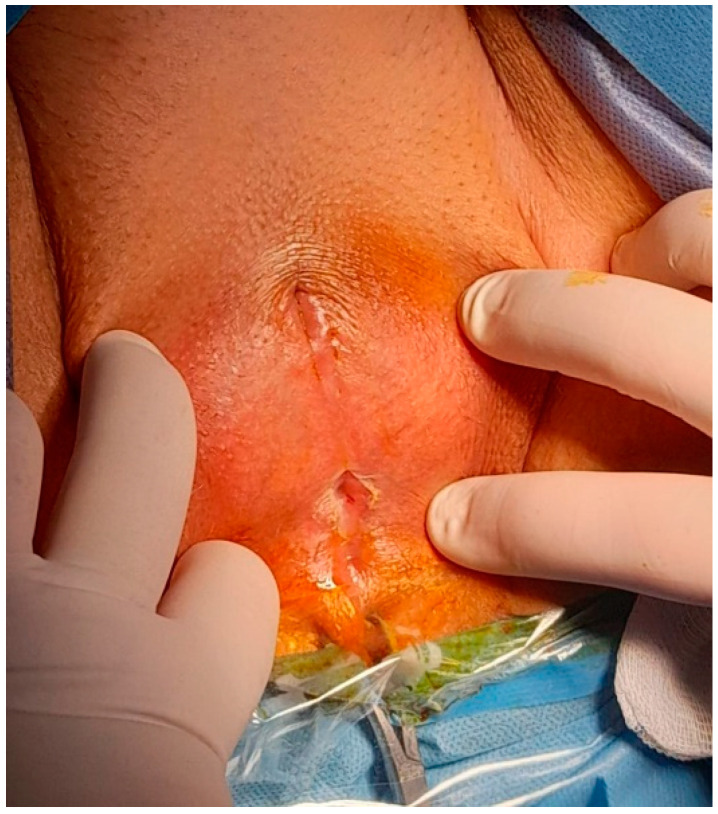
Complete labia majora adhesion with a small posterior opening.

The patient described was an 84-year-old postmenopausal woman who presented with nearly complete closure of the vaginal introitus due to the fusion of the labia majora. Her primary complaints included dysuria and intermittent urine leakage. The constant moisture in the vulvar region caused by dribbling urine resulted in irritation, leading to the progression of the labial adhesion to its complete form. Upon clinical examination, there was only a small opening of approximately 5 mm at the posterior fourchette, sufficient for urine to escape drop by drop. Additionally, the anterior fusion was accompanied by the partial disappearance of the clitoral hood, which is the skin covering the clitoris (Figure 1).

The patient reported that her last menstrual period occurred 40 years ago, indicating early menopause at age 44. She stated that she had been sexually inactive for more than 25 years. Her obstetric history included seven pregnancies, one vaginal birth, and six induced abortions. The patient’s comorbidities included hypertension for 46 years, type II diabetes for approximately one year, viral hepatitis C diagnosed in 2020, and chronic venous insufficiency.

Regarding clinical history, the patient initially presented with symptoms of dysuria four years before the first surgical intervention. Despite undergoing urethral dilations with bougies in 2020, there was no clinical improvement. At that time, labial coalescence extended from the introitus upwards.

**Figure 2:**
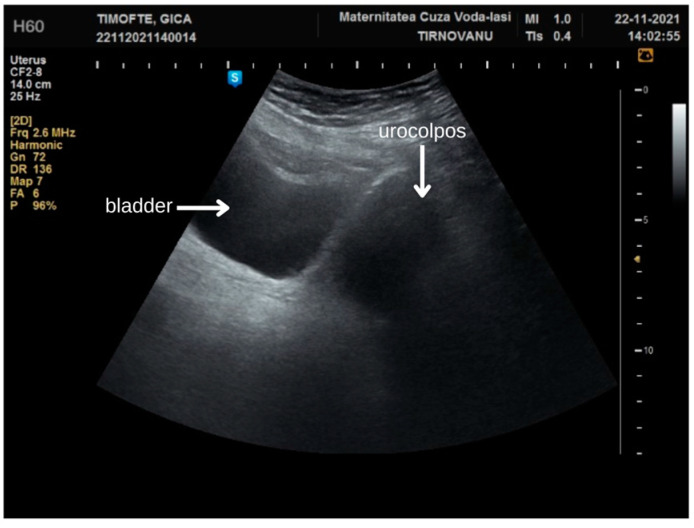
Ultrasound at first admission for labial fusion.

Her surgical history was significant, including a procedure in 2021 for complete fusion of the labia majora, approximately two and a half years prior to the current admission. During that hospitalization, she experienced complete urinary retention for about three days. This symptom was attributed to the accumulation of urine in a blind pocket formed by the adhesions, which would subsequently leak onto her undergarments post-voiding. During the surgical procedure, a urinary tract infection with *Escherichia coli* was identified via bladder catheterization. The anatomical region affected by the synechia had formed a pocket, predisposing it to urine retention and microbial proliferation. In the middle third of the labial coalescence, a 5 mm membrane allowed for urine to exit during abdominal compression. An abdominal ultrasound examination revealed urine in both the bladder and the urocolpos (Figure 2), along with bilateral hydronephrosis classified as grade I. We successfully inserted a urinary catheter into the vagina, which resulted in the discharge of 1.5 L of urine.

After the initial surgery, which successfully opened the vaginal introitus, the patient was prescribed topical estrogen therapy that is typically recommended for a limited duration (4–6 weeks). In this case, we suggested the application of topical estrogen twice weekly for six months, starting three weeks postoperatively, given the recurrence of the condition. At the postoperative follow-up two months later, the vulva appeared to be normal. The patient was instructed to perform regular dilation, with specific guidance provided on the technique and frequency. Additionally, we recommended the long-term application of a mild ointment following daily warm water soaks, along with gentle traction to minimize the risk of recurrence. Unfortunately, the patient did not come for follow-up, but only for the reappearance of the labial coalescence.

Upon her first admission to the hospital, the patient reported a recurrence of the coalescence that had persisted for about four months, occurring two years and six months after the primary surgery.

Laboratory blood analysis on admission revealed a normal value of white blood cells (WBCs) close to the superior limit (9.17 × 109/L), without neutrophilia (neutrophils 6.32 × 109/L) and anemia (hemoglobin 11.7 g/L), a normal value of blood glucose level (107 mg/dL), and elevated creatinine (1.48 mg/dL) and urea (85 mg/dL).

**Figure 3:**
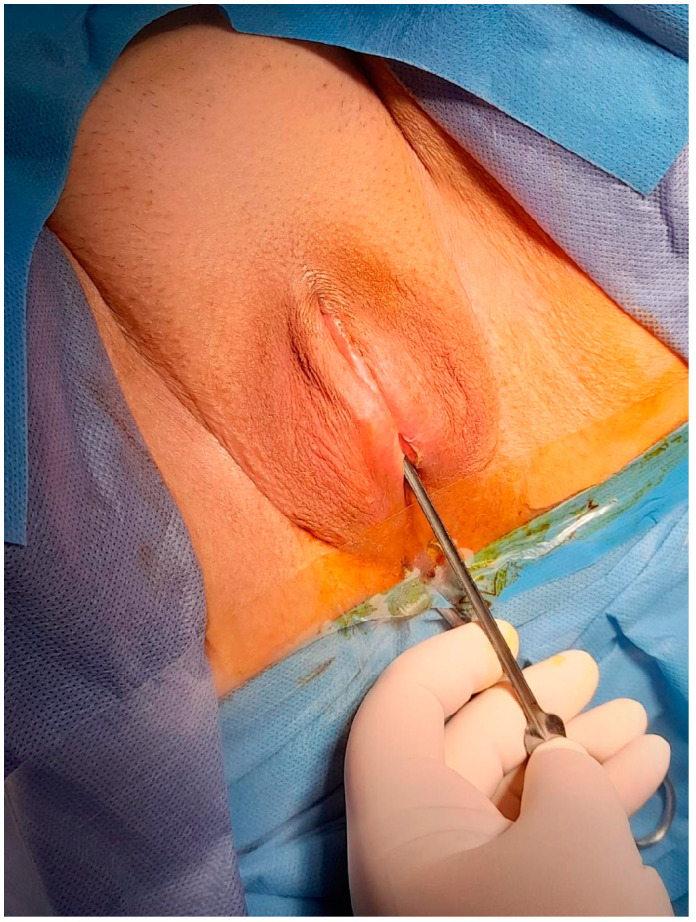
Introduction of a buttoned stiletto into the small opening in the fused labia majora.

**Figure 4:**
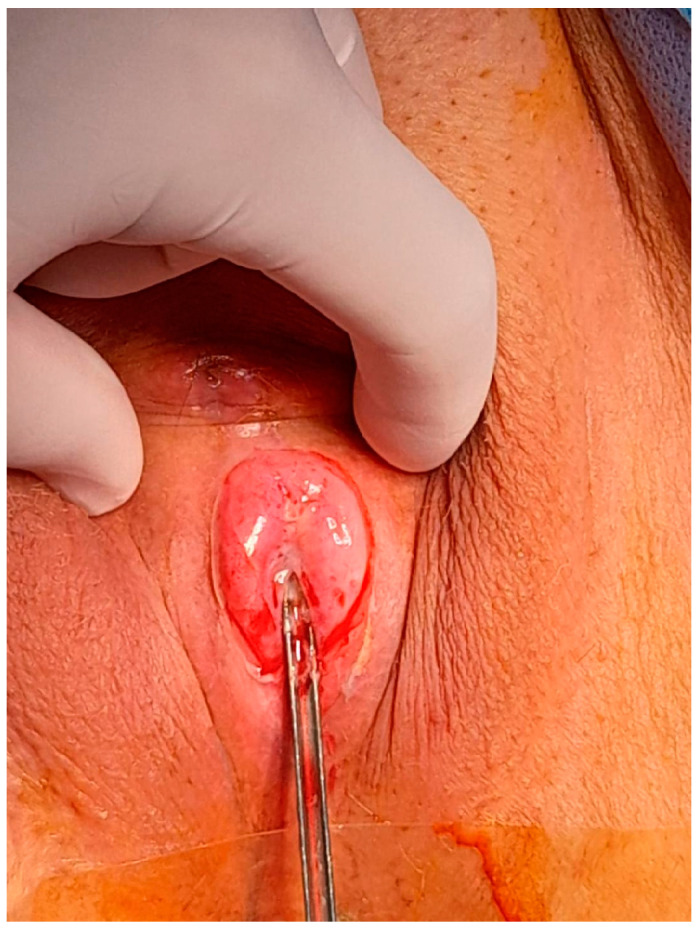
Sectioning the upper portion of the fusion with the protection of the urinary meatus.

**Figure 5:**
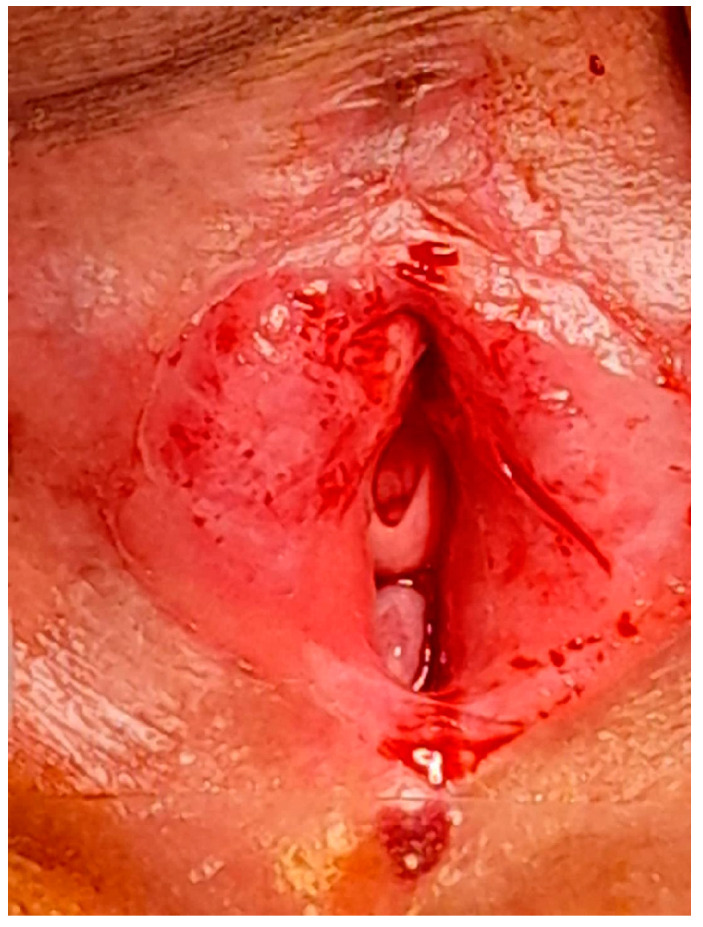
The vaginal introitus and the urethra clearly visible.

After obtaining informed consent, the patient was taken to the operating room. Under spinal anesthesia, she was positioned in lithotomy and prepared for the procedure. A small opening in the fused labia majora was elevated, and a midline vertical incision was made upward from the posterior orifice using a buttoned stiletto, clearly exposing the vaginal introitus up to the external urethral orifice (Figure 3, Figure 4 and Figure 5). The adhesion between the labia minora and the corresponding labia majora resulted in the resorption, shrinkage, or complete disappearance of the labia minora (Figure 5). The vaginal epithelium appeared to be atrophic and inflamed as a secondary effect. A 14F catheter was used to drain the bladder, and urinalysis revealed a urinary tract infection caused by *Escherichia coli*.

**Figure 6:**
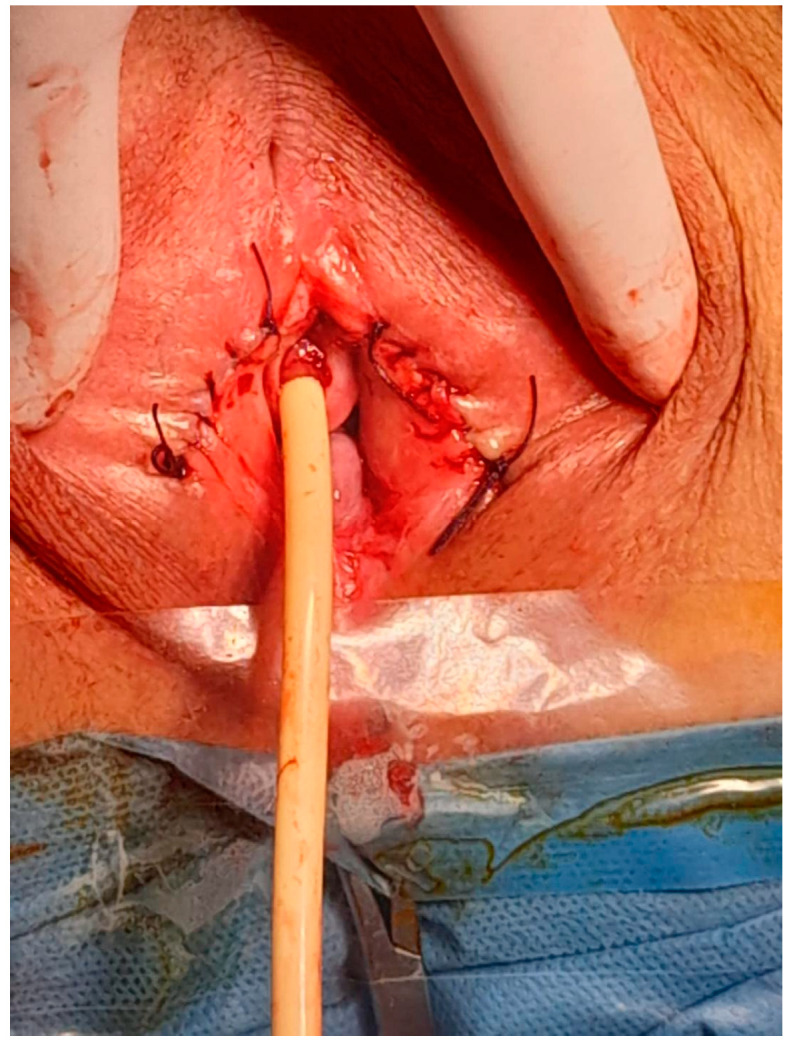
Suture in order to close the exposed area in the labia majora.

**Figure 7:**
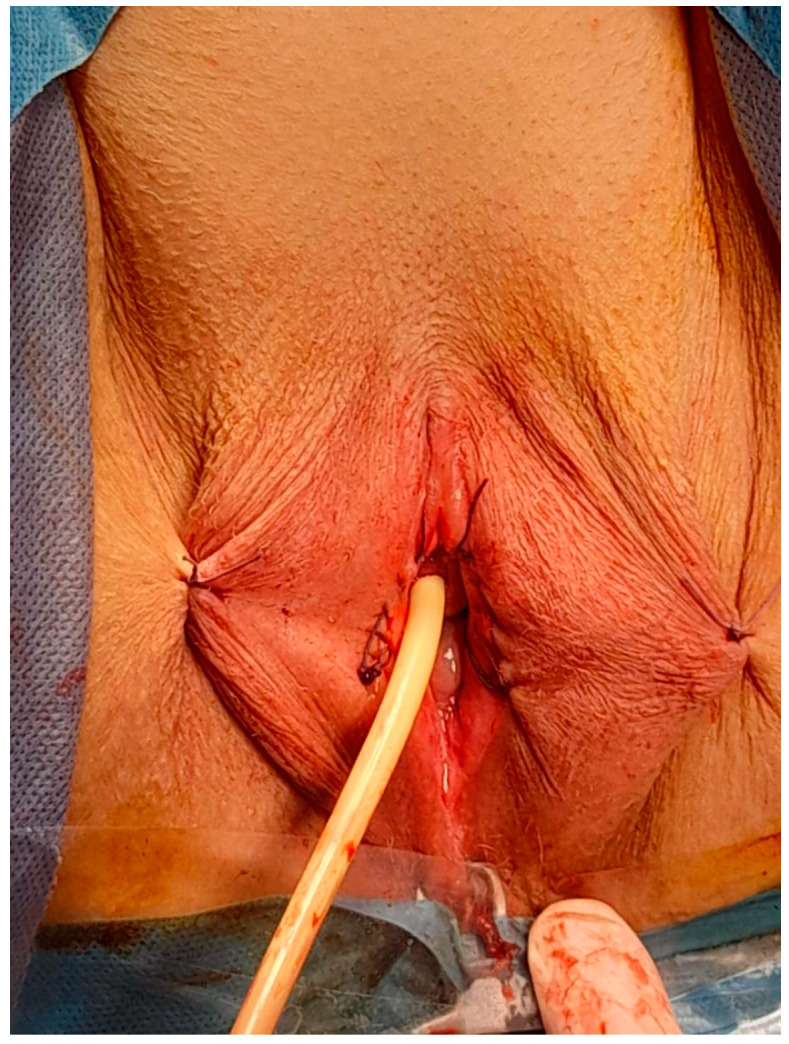
Vulvar appearance at the end of the surgical procedure.

The mucosa and the skin were sutured with surjet 2-0 Vicryl (Figure 6). We fixed both major labia on the perineal skin to keep the introitus open (Figure 7).

The Foley catheter was removed after seven days to prevent urine from encountering the vulvar wound while the patient was on antibiotics. Following this, she was able to urinate without difficulty and had a smooth postoperative recovery. The patient was discharged from the hospital eight days after the surgery and was prescribed topical estrogen for one month.

**Figure 8:**
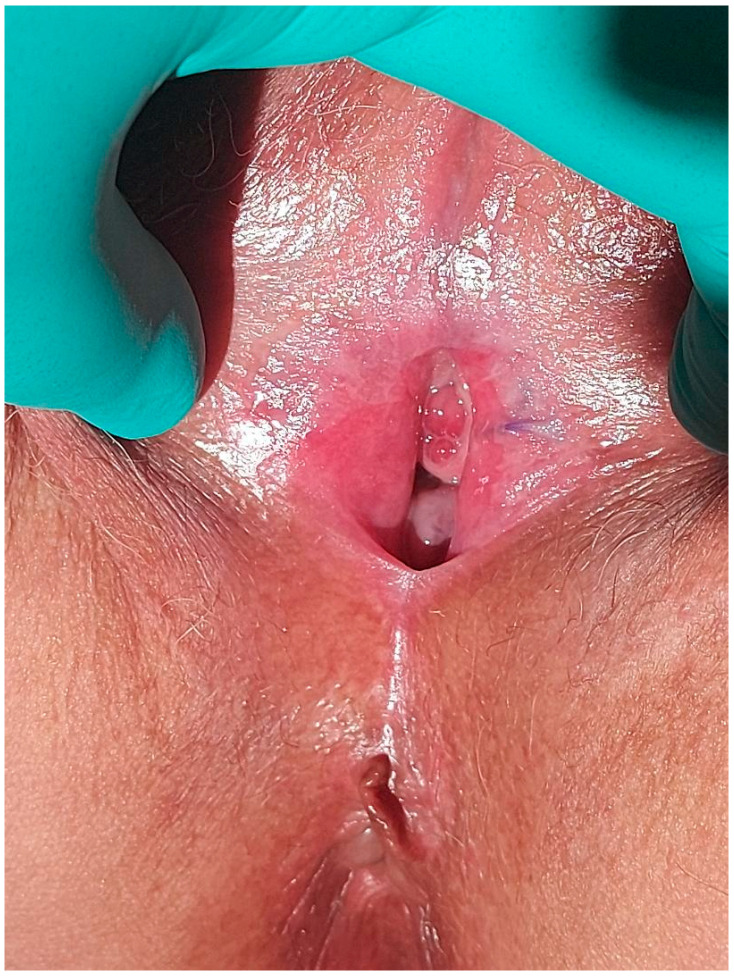
The aspect of the vulva three weeks after surgery.

Three weeks following her discharge, she returned for evaluation (Figure 8) and reported normal urination. We recommended to the patient the use of estrogen cream topically twice a week for six months. Due to the short recurrence interval, we opted for follow-up evaluations every six months to monitor for further relapses and adjust management as needed. We advised also the long-term application of a bland ointment following daily warm water soaks and gentle traction to minimize the risk of recurrence.

## 3. Discussion

Labial fusion is a rare condition in postmenopausal women, being much more common in prepubescent girls. Most of the cases documented in the literature, similar to ours, involve early menopause and a prolonged absence of sexual activity [6,7].

It is known that labial fusion can also occur in the absence of known risk factors. However, the exact causes of recurrence remain poorly understood and should be further investigated [8,9]. A low-estrogen environment may contribute to the formation of adhesions by reducing the inhibition of fibroblasts, potentially leading to fibrosis [8,9]. Additionally, factors that promote genital adhesions, such as poor hygiene, chronic irritants, and recurrent vaginal infections, should be addressed [10]. In elderly women, particularly those immobilized for extended periods due to fractures, these risk factors must be minimized to prevent labial coalescence. In our patient, there was no history of vulvar lesions, repeated infections, chronic inflammatory conditions, or local trauma but only atrophy.

For diabetic patients, their blood sugar levels should be carefully managed to reduce the risk of recurrence [11]. Although diabetes is considered to be a risk factor for labial adhesion, in this case, the patient had recently been diagnosed with diabetes, which was controlled with medication (glycemia 107 mg/dL).

Although the symptoms can range from mild discomfort to severe urinary flow obstruction, the impact on a patient’s quality of life should not be underestimated. The embarrassment that postmenopausal women feel when discussing their condition can result in the progression to severe labial lesions. Those patients with labial adhesions often experience difficulty in voiding, or urinary incontinence [12,13]. When urine flow is obstructed, it can result in urinary retention and recurrent urinary tract infections [12]. In our case, the patient consistently presented with difficulty voiding and, at the time of the first surgical intervention, urinary retention. The retention occurred because the orifice in the labial fusion was positioned higher, causing urine to stagnate in the vaginal cavity.

Renal function tests and imaging of the genitourinary system may be necessary to identify any underlying issues, particularly in cases presenting obstructive symptoms [14]. Early diagnosis and treatment of labial fusion and genital adhesions are therefore crucial. In our patient, the renal function tests were elevated before the surgery but returned to normal levels two days post-surgery.

Histologically, labial adhesion presents with a thin, atrophic epithelium, primarily due to the low-estrogen environment associated with the prepubertal or postmenopausal states. This epithelium is often devoid of the typical keratinized layer and lacks robust glycogen stores, making it more fragile and prone to microabrasions and inflammation. The underlying connective tissue appears to be more compact and less vascularized, with reduced collagen and elastin fibers, contributing to rigidity in the affected area [15].

In cases of labial fusion, there may be a mild inflammatory infiltrate at the junction of the fused labia, often composed of lymphocytes, which may indicate low-grade chronic inflammation. This inflammatory response may exacerbate adhesion by promoting fibrosis and the formation of a thin fibrotic band that physically connects the labial surfaces. Additionally, there may be sparse capillaries and reduced mucosal glandular activity, which lead to dryness and further susceptibility to adhesion and fusion [16].

Estrogen helps to restore the epithelium and maintain tissue health, but the long-term effectiveness of this approach varies. Some studies suggest that recurrence may occur despite estrogen treatment, highlighting the need for complementary strategies or adjusted hormone dosages [8,9]. In postmenopausal women, topical estrogen cream is less effective than in pediatric patients, and there are insufficient data on the use of betamethasone for treating labial fusion [9]. In our case, only the topical estrogen treatment was not successful after the first surgery, with the recurrence of labial coalescence.

Several factors influence the choice of treatment, including the patient’s age, the severity of the symptoms, the presence of other concomitant conditions, and the extent of the fusion. In such cases, surgery is typically considered to be the first-line and definitive treatment option. In cases of complete bladder outlet obstruction, surgical treatment is unavoidable to restore normal urinary tract function [8,17]. However, the choice of surgical technique must be tailored to the severity of the fusion and the patient’s overall condition, considering the postoperative risks and the likelihood of recurrence [18,19]. New surgical methods combining Z- and Y-V-plasty have been developed to effectively treat severe labial adhesions in postmenopausal women, resulting in no recurrence after a few months. This approach offers a promising alternative for managing severe cases of labial adhesion in postmenopausal patients, with a reduced risk of recurrence [20].

Recurrence of adhesions occurs in 14–20% of patients following surgery. Emphasizing the importance of topical estrogen application and regular digital separation of the vulva, especially in sexually inactive patients, is crucial [9,21]. In recent years, new methods have been introduced to prevent the recurrence of genital adhesions, including treatments such as genital platelet-rich plasma (PRP), vaginal laser therapy, and radiofrequency [8,9]. These treatments are administered in regular sessions, with the frequency and duration tailored to the patient’s specific condition. Our patient had a prior diagnosis of labial fusion, and, although she recalled undergoing a successful surgical separation with an introitus of 3 cm, the condition recurred. This time, if we observe a tendency for relapse during the follow-up, we might consider recommending the use of a vaginal dilator.

There are limited data but emerging interest regarding labial adhesion in the context of underlying systemic conditions. For instance, postmenopausal women or individuals with systemic inflammatory diseases may present with labial adhesions in conjunction with joint stiffness or musculoskeletal complaints. This may be partly due to the chronic inflammation and tissue degeneration that affect both the connective tissues in joints and the labial epithelium, especially under low-estrogen conditions [22].

Orthopedic conditions such as severe hip joint disease can significantly contribute to the development of this condition. When hip joint disease limits movement, it can impede adequate perineal hygiene. This limited mobility often leads to the excoriation (damage or irritation) of the skin around the vulva due to difficulty in cleaning, especially in cases where women may have trouble with abduction, flexion, and internal rotation of the hip joints. Moreover, osteoarthritis or hip deformities can reduce joint flexibility, making daily hygiene practices more challenging. The resulting inadequate hygiene can promote bacterial accumulation and inflammation, irritating the already estrogen-deficient vulvar tissue common in postmenopausal women. This irritation, combined with decreased estrogen levels that lead to the thinning and increased fragility of the vulvar and vaginal epithelium, create a favorable environment for labial adhesion formation. Moreover, physical discomfort from hip conditions often limits sexual activity, a natural preventive factor against labial fusion. The mechanical restrictions imposed by the hip disease also make it challenging for women to maintain the positions necessary for intercourse, which might otherwise help to keep the labia separated. Consequently, the adhesions become more likely to develop and persist, especially in sexually inactive postmenopausal women who are dealing with hip joint disease [5].

Additionally, in some pediatric cases with labial fusion, underlying conditions such as congenital skeletal anomalies or connective tissue disorders may predispose to both labial and orthopedic issues due to shared connective tissue weaknesses. Estrogen deficiency, common in conditions of delayed puberty or hormonal imbalances, can impact bone density and joint health while simultaneously increasing susceptibility to epithelial atrophy and adhesions [23]. Moreover, autoimmune diseases may heighten the risk of adhesions through chronic inflammatory mechanisms that impact both mucosal epithelia and other connective tissues. In pediatric cases, hormonal imbalances and dermatologic conditions, such as atopic dermatitis, can increase the risk of labial adhesion due to a predisposition to localized inflammation and recurrent irritation [15].

Labial adhesion can also be associated with various endocrine disorders, particularly those that affect hormonal levels. Conditions such as polycystic ovary syndrome can lead to irregular menstruation and altered estrogen levels, potentially increasing the risk of labial fusion. Furthermore, hypothyroidism, characterized by decreased metabolic function and hormonal imbalances, may contribute to skin and mucosal changes, making the labial tissues more susceptible to adhesion [16]. These endocrine disruptions may lead to an overall decrease in tissue integrity and resilience, facilitating the development of labial fusion.

As stated before, metabolic disorders, such as diabetes mellitus, may also play a role in the occurrence of labial adhesion. Patients with poorly controlled diabetes often experience changes in skin and mucosal health, including increased dryness and susceptibility to infections. Elevated glucose levels can lead to a glycation process that affects tissue function and integrity, potentially contributing to adhesion formation. Moreover, diabetes-related complications, including neuropathy and reduced sensation, may lead to delayed recognition and treatment of labial adhesions, exacerbating the condition [10].

Infectious diseases can significantly impact the development of labial adhesion, particularly those that cause chronic inflammation or irritate the vulvar area. Conditions such as recurrent vulvovaginal candidiasis or bacterial vaginosis may lead to inflammation of the labial tissues, promoting a favorable environment for adhesion formation. The inflammatory response associated with these infections can result in edema and subsequent fibrosis, ultimately leading to labial fusion. Additionally, sexually transmitted infections may cause significant irritation and scarring, further contributing to the development of adhesions if not properly managed [16].

Certain neurological disorders may also be linked to labial adhesion, especially those that affect sensation and mobility. Conditions like multiple sclerosis or cerebral palsy can lead to altered sensory perception in the vulvar region, resulting in inadequate care and hygiene. Patients may be less aware of irritations or infections, increasing the risk of developing labial adhesion due to ongoing inflammation or injury. Furthermore, neurological impairments can complicate the ability to seek a timely medical intervention, resulting in more severe cases of labial fusion [24].

Labial fusion has been identified in patients following allogeneic hematopoietic cell transplantation, often as a complication associated with chronic graft-versus-host disease (cGvHD). In those cases where women developed labial fusion post-hematopoietic cell transplantation, cGvHD—especially in its sclerotic form—appeared to be a significant contributing factor, affecting the genital region and resulting in adhesions between the labia minora. These patients must be asked about dyspareunia and dysuria. Those with genital symptoms warrant referral to a gynecologist. Scrivani et al. published a series of five cases of labial fusion occurring after matched allogeneic peripheral blood hematopoietic cell transplantation [25].

These associations suggest that evaluating and managing labial adhesions should involve a multidisciplinary approach, addressing both local treatment and any contributory systemic factors.

## 4. Conclusions

Surgical intervention remains the primary treatment option for complete labial fusion in postmenopausal women, particularly in cases involving bladder outlet obstruction or significant urinary symptoms. While surgery effectively alleviates the obstruction, the potential for recurrence is a notable concern. The postoperative use of topical estrogen creams is commonly recommended to restore mucosal integrity and address the underlying estrogen deficiency, thereby reducing the risk of recurrence. However, despite hormonal therapy, some patients may still experience recurrences, highlighting the importance of long-term follow-ups and individualized treatment plans.

## Data Availability

Data are available on personal request.
